# Mesenchymal stem cells to treat female infertility; future perspective and challenges: A review

**DOI:** 10.18502/ijrm.v20i9.12061

**Published:** 2022-10-10

**Authors:** Yasmeen Saeed, Xiaocui Liu

**Affiliations:** ^1^Guangdong Provincial Key Laboratory of Utilization and Conservation of Food and Medicinal Resources in Northern Region, Shaoguan University, Shaoguan City, Guangdong Province, China.; ^2^Guangdong VitaLife Biotechnology Co., LTD, Foshan, Guangdong, China.

**Keywords:** Pregnancy, Infertility, Female, Stem cell transplantation, Uterine diseases, Mesenchymal stem cells.

## Abstract

Infertility negatively impacts the overall health and social life of affected individuals and couples. Female infertility is their inability to perceive pregnancy. To date, polycystic ovary syndrome, primary ovarian insufficiency, fallopian tube obstruction, endometriosis, and intrauterine synechiae have been identified as the primary causes of infertility in women. However, despite the mutual efforts of clinicians and research scientists, the development of an effective treatment modality has met little success in combating female infertility. Intriguingly, significant research has demonstrated mesenchymal stem cells as an optimal source for treating infertility disorders. Therefore, here we attempted to capsulize to date available studies to summarize the therapeutic potential of mesenchymal stem cells in combating infertility in women by focusing on the underlying mechanism through which stem cells can reduce the effects of ovarian disorders. Furthermore, we also discussed the preclinical and clinical application of stem cell therapy, their limitation, and the future perspective to minimize these limitations.

## 1. Introduction

Infertility affects 8-12% of couples and has become a common problem worldwide (1-3). According to the epidemiological categorization, a female is considered infertile if she remains unsuccessful even after several attempts to be pregnant (4, 5). An approximate estimation for the prevalence of overall infertility has indicated that 33-41% of infertility could be due to female cause, while male causes contribute 25-39%, and mixed causes are about 9-39% (6). The statistics reveal that women's infertility is among the most significant factors (7) due to its increasing epidemiology, affects millions of females worldwide (7, 8). Accordingly, ovulation disorders, i.e., hypothalamic dysfunction, primary ovarian insufficiency, or polycystic ovary (PCOS), are considered the major cause of female infertility. At the same time, endometriosis and tubal infertility are also among the preeminent cause of female infertility (7). The ovary is a complex and highly regulated organ where minor dysregulation can ultimately lead to infertility (9). While ovulation and hormonal testing are more commonly used methods for diagnosing female infertility.

Moreover, further advent in technology has introduced some assisted reproduction strategies i.e., intrauterine insemination, and in-vitro fertilization to minimize the damaging effects of ovarian dysfunction (1, 10-12). Other available treatment options include the intervention of supplements or antioxidants, such as zinc, vitamin E, and L-carnitine. Despite their frequent application, these treatment strategies have shown their limitations and side effects (1, 13). Hence, to date, there is a darn lack of an effective treatment strategy for curing female infertility (14). Therefore, researchers and clinician have been investigating more efficient and novel therapeutic measures for infertility. Among these strategies, stem cell therapy holds significant importance, being undifferentiated cells, their self-renewal for longer duration without developing any change (7). Nonetheless, as compared to other group of stem cell, mesenchymal stem cells (MSCs) have been indicated to possess advantages over other types of stem cells for being free of ethical concerns and teratoma formation (7, 15).

Herein we will discuss the published studies about the cause of infertility, to date available therapies, and application of MSCs to treat the infertility in women.

## 2. Prevalence and etiology of female infertility 

Infertility is defined as primary and secondary infertility; the primary is attributed to a woman who never perceives pregnancy, while secondary infertility indicates a woman who perceives one successful pregnancy but later becomes incapable of perceiving pregnancy (14). The most common prevalence of primary infertility occurs in developed countries, while secondary infertility has been found as a major cause of infertility in developing countries (16). The other reason for infertility could be attributed to the presence of oocytes in a limited defined number (14) which naturally tend to decline with aging and ultimately enhance the risk of miscarriage (17). In addition to the abnormality of reproductive organs, the other major reason for female infertility is a disturbance of the central nervous system that controls the secretion of hormones in the reproductive system (14) further suggest the correlation between infertility and endocrine disorders that is attributed with the hormonal imbalance in the reproductive system. Though several factors attributed to female infertility remained unexplained to date (18), major research have reported the association of infertility with the complex intrinsic events which build on various factors such as the environmental or genetic factors, the age of patient, and etiology (1). Therefore, it is important to consider these factors to develop an efficient therapeutic strategy. However, considering the complexity of underlying signaling pathways of reproductive system disorders multiple molecular factors could be involved which not only cause ovarian dysfunctions but also impact individual's overall systemic health (9, 19). However, to date, the major factors and comprehensive mechanism with peculiar biomarkers that result in infertility remains unknown. Therefore, not a single efficient or effective treatment is available to inhibit the causes of female infertility disorder. Further, we will brief on the currently available treatment.

## 3. Currently available combat tactics for infertility fighting

As reported in the previous section, endocrine system disorder is among the major contributing factor to female infertility. Therefore, to date, hormone replacement therapy has been widely implicated to treat various types of infertility disorders. For instance, Clomid (a widely used gonadotropin) has been reported to trigger an excess generation of luteinizing hormone and follicle-stimulating hormone to be released by the pituitary gland, which induce the ovulation and promotes follicular growth (20) and ultimately results in the production of multiple eggs (1, 21). However, this therapeutic strategy carries the risk of breast cancer (22, 23). The other treatment option is fertility drugs to treat ovulation disorders (1). However, these drugs have exhibited some setbacks, such as preterm birth, ovarian hyperstimulation syndrome, multiple births, and ovarian tumor (20). Though surgical therapeutics strategies, i.e., assisted reproductive technologies, ovulation induction, hysteroscopic, laparoscopy, and fallopian tube surgeries (1), and superovulation have shown efficiency in optimizing the condition for perceiving pregnancy. However, the efficacy and safety of these strategies have not been completely evaluated (7), and the outcome of these strategies also remained unsatisfactory.

Nonetheless, despite the advancement in treatment strategies for fertility disorders, the overall percentage of infertility remained more than 80% (24). While the development of an effective therapeutic strategy not only requires considerable attention to the physical, psychological, economic, and time-related factors but also needs to apply the novel technology at the cellular level to understand the underlying molecular mechanism completely. Hence, it is essential to establish an alternative and novel therapeutic modality to cure female infertility. While despite being in their infancy, regenerative medicines offers a promising therapeutic candidate, a future set of comprehensive study is required for its clinical application. Accordingly, further, there will be a discussion about the function, applications, and limitations of stem cell therapeutic strategies.

## 4. Stem cell therapy in treating female infertility and significance of using MSCs

Based on the preclinical study data, stem cell therapy is the most promising candidate for treating infertility disorder. At the same time, further insight into its differentiation potential characterized stem cells as either pluripotent or multipotent. However, some studies have indicated that pluripotent stem cells or embryonic stem, i.e., germ-line stem cells or ovarian surface epithelial cells, can be used as cell sources for neo-oogenesis and oogenesis (25). Such as, an interesting recent finding has revealed the immunomodulatory properties of embryonic stem cells and demonstrated that by altering the gene expression profiles of mesenchymal stem-like cells, human embryonic stem cells (hESCs) can be generated from both normal (diploid) and abnormal (triploid) hESCs lines (26). However, due to an incomplete understanding of the major molecular events of oogenesis, i.e., follicle formation and meiosis, these results could not be reproduced in-vivo culture system (25). Hence, despite having a good experimental evidence, it is not ethically possible to use embryonic stem cells in a clinical setting, largely due to its risk of developing cancer.

However, on the other hand, MSCs, that are categorized as multipotent adult stem cells, have been extensively applied for research and clinical use due to their advantage of differentiation into multiple lineages of tissues/cells and having no ethical issue for its application (27-28). Thus, recent years have seen remarkable advancement in the clinical application of MSCs due to their safety and high efficacy against various disorders (29-32). For instance, MSCs therapy has been reported to positively affects chemotherapy-induced lesions, premature ovarian failure, and PCOS (33-37). Besides, the underlying mechanism indicates that stem cells can be used therapeutically for a variety of ailments that is attributed to the secretion of various mediators particularly cytokines that could effectively modulate the inflammatory and immune activation processes that significantly reduce inflammation-associated tissue damage (38-40). Hence, MSCs aid in the reduction of ovarian damage by targeting inflammation.

Nonetheless, it is also important to note that most of the above-mentioned research has been carried out using the rodent's model, which significantly differs from humans, particularly regarding the female reproductive system. Collectively, the above-mentioned studies suggest that MSCs therapy can provide longer reproductive life in larger animals, peculiarly, cattle that are more identical to humans, and present an economically admissible model (30). Accordingly, some latest preclinical trial studies and their outcome have been summarized (Table I). While preclinical trials and their application in large farm animals remained under consideration (41, 42). In the next section, we will discuss the various sources of stem cells and their role in the treatment of female infertility in light of previously reported studies.

**Figure 1:**
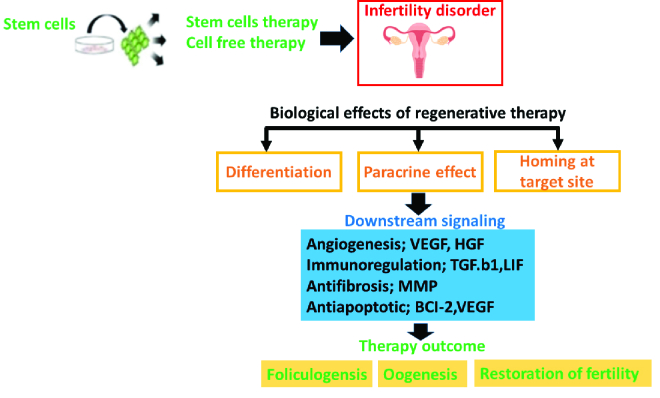
Schematic presentation of general overview of the effects of mesenchymal stem cell (MSC) based cell therapy for infertility disorders by demonstrating the downstream signaling factors. Shortly, graphic presentation theoretically indicates that after exposure to the stem cell-based therapy or cell-free to ovarian or endometrial disorder, its biological effects (i.e., paracrine factors, differentiation, and migration to the target site) effectively aid in reducing the deleterious effects by aiding in folliculogenesis, oogenesis and ultimate recovery due to the production of vital downstream signaling factors.

Although various types of MSCs, such as bone marrow-derived MSCs (BM-MSCs), umbilical cord-derived MSCs (UC-MSCs), and endometrial derived MSCs (EnMSCs) have been reported to demonstrate significant therapeutic efficiency against infertility, however, regarding female infertility, some unusual and less researched stem cell lines such as peritoneum MSCs (PeMSC, which is derived from peritoneum mesothelium consisting of intraperitoneal or intestinal space) have been found to differentiate into ovarian cell-like cells (43). While another study has demonstrated the positive outcome of PeMSCs in clinical trials (44). Accordingly, we have presented a schematic presentation of a general overview of the effects of MSCs based cell therapy for infertility disorders in figure 1.

Hence, collectively stating that despite having the unique ability to restore the quality and quantity of oocytes MSC possesses, there is a lack of clinical evidence that could prove and validate the treatment of MSCs as a safe clinical therapeutic option for infertility disorders (30). Hence, the further sections, based on previously reported studies, will briefly highlight the role of a few important MSCs, i.e., UC-MSCs, BM-MSCs, and EnMSCs cell lines that have been widely reported in the recent literature for the treatment of infertility and have shown promising outcome to suggest a future set of study. This presented the cell-free therapeutic approach to treat PCOS, or maybe other poor-quality oocytes. Accordingly, we have summarized this few recent preclinical research in table I.

**Table 1:** Summary of recently reported preclinical trials using stem cell therapy to treat female infertility


**Author, yr (Ref)**	**Types of models**	**Source of stem cells**	**Time of administration**	**Dose**	**Delivery route**	**Efficacy**	**Important findings**
** Xie ** ***et al.*** **, 2019** **(35)**	PCOS in C57BL/6 mice was induced with DHEA	hUC-MScs	21 days after the modeling	(2 × 10^6^ passage 4 hUC-MSCs suspended in 0.2 ml normal saline	Tail vein injection	By inhibiting local (ovaries and uterus) and systemic inflammatory responses, hUC-MSC treatment effectively improved the pathological changes and function of PCOS in mice	A mouse model of DHEA-induced PCOS was alleviated by MScs by inhibiting inflammation
** Kalhori ** ***et al.*** **,** ** 2018 (45)**	NMRI mice PCOS was induced through daily subcutaneous injections of testosterone enanthate dissolved in sesame oil	BM-MSCs	At 1 ${}^{\text{st}}$ and 14 ${}^{\text{th}}$ day after experimental induction of PCOS	(10^6^ MSCs/animal)	Injected into the mice through the tail vein	The number of oocytes increased significantly as folliculogenesis increased whereas the number of primary and preantral follicles decreased significantly	Polycystic ovaries in mice injected with BM-MSCs showed improved folliculogenesis, oocyte quality and endocrine function due to their antioxidant, anti-inflammatory and anti-apoptotic properties
** Jafarzadeh** ***et al.*** **, 2018 (46)**	PCOS, 21-day-old female NMRI mice received daily subcutaneous injections of 6 mg/100 g bodyweight dehydroepiandrosterone	hBM-MSC derived CM	Condition medium was exposed to oocytes at the GV stage	The cells in the third passage were seeded at a density of 1 × 10^4^ cells/cm^2^. Then, the CM was concentrated 10 times (10 × ) by centrifugation	Condition medium was exposed to oocytes at the GV stage	hBM-MSC-CM improved the oocyte -IVM, cytoplasmic maturation, fertilization and early embryo development rates	This study suggest that the cell-free therapeutic strategy can be efctive in the treatment of PCOS
** Chugh ** ***et al.*** **,** ** 2021 (47)**	For in vitro experiment, conditioned media from BM-hMSC was exposed to androgen-producing H293R cells and analyzed androgen-producing gene expression. For in vivo experiment, BM-hMSC were implanted into a letrozole-induced PCOS mouse model	hBM-MSCs	5 wk after modeling	5.0 × 10^5^ cells per ovary resuspended in 10 μl PBS	Intra-ovarian injection of BM-hMSC via laparotomy	This research indicated the efficacy of intra-ovarian injection of secretome of BM-hMSC that effectively improved the PCOS-related symptoms	BM-hMSC or its secretome potentially reversed the inflammation caused by PCOS-via IL-10 secretion
PCOS: Polycystic ovary syndrome, DHEA: Dehydroepiandrosterone, hUC-MScs: Human umbilical cord-mesenchymal stem cells, BM-MSCs: Bone marrow mesenchymal stem cells, hBM-MSC: Human bone marrow mesenchymal stromal cells, CM: Condition medium, GV: Germinal vesicle, IVM: In vitro maturation, PBS: Phosphate buffered saline, IL-10: Interleukin-10							

### UC-MSCs and their underlying mechanism for fertility disorders

The most prominent properties of UC-MSCs that make these cells a reliable option are easy to collect, self-renewal, and lower risk of tumor formation (48). According to previously reported study UC-MSCs can repair the premature ovarian failure by reducing the apoptosis in ovarian cell which exhibited the improvement in the function of ovary in animal model as well as in human trials (49-51). Besides, accumulated studies attempted to unravel the underlying mechanism which aid UC-MSCs in diminishing the damaging effects of ovarian disorders. Though number of signaling mechanism have been reported to date, however, the mitogen-activated protein kinase signaling pathway and insulin signaling pathway have been found to possess central role in treating the impact of ovarian damage (52). Moreover, UC-MSCs play their role in angiogenesis by stimulating the secretion of vital growth factors such as placental growth factor, transforming growth factor-beta 1, vascular endothelial growth factor, and hepatocyte growth factor (53). Moreover, UC-MSCs have also been reported to control fibrosis and enhance cell proliferation by upregulating the expression of vascular marker's which aid in alleviating the inflammation (54).

For instance, it has been found that UC-MSCs can overhaul the injured endometrium in the rat model. Intriguingly, UC-MSCs have been also been reported to overturn apoptosis in ovarian cells either through customary adjustment of the tunica albuginea and surface epithelium or by downregulating the caspase-3 and elevation in the expression of proliferating cell nuclear antigen, transforming growth factor-beta, and cytokeratin 8/18 (55). Furthermore, studies have illustrated that UC-MSCs could aid in the recovery of perimenopausal rats by accelerating the release of cytokines (53). At the same time, another study found that intramuscular injection of UC-MSCs can alleviate the marring effects on human endometrial cells (56). Importantly, a phase-I clinical trial has stated that collagen scaffolds containing UC-MSCs can effectively restore endometrial differentiation, vascularization, and proliferation by elevating the estrogen receptor α and angiogenic factors (57). These studies indicates UC-MSCs as the most reliable and widely used stem cells for not only research purpose to treat female infertility but also for preclinical and clinical trials; however, further clinical evidence is a prerequisite for their clinical application (9).

### BM-MSCs

Stem cells derived from bone-marrow have been extensively used for multiple therapeutic purposes, yet its clinical application for the treatment of infertility in women remained elusive to date. Although, a previously reported study has used mice model of PCOS and found the improvement in endocrine function, oocyte quality, and folliculogenesis due to the anti-inflammatory, anti-apoptotic, and anti-oxidative properties of PCOS (45). Another interesting recent evidence from the larger animal has indicated that the autologous BM-MSCs isolated from juvenile macaques significantly restore the ovarian structure by reduction of cell apoptosis and aid in the regeneration of blood vessels and follicles which ultimately inhibited the fibrosis and prevent the ovarian aging (58). Besides, chemotherapy is considered one of the significant causes of infertility in female cancer survivals, which results in a drastic reduction of primordial follicles and ultimately causes premature ovarian failure. Moreover, another study has indicated that aged women (with history of being exposed to chemotherapy) after intra-ovarian administration of BM-MSCs exhibited the significant restoration of ovarian function (42). However, a comparative study suggested that ovarian stromal cells could significantly promote the maturation of ovarian follicle in the damaged ovary as compared to the BM-MSCs (59). However, there is a lack of data to support these findings. Thus, speculating that regarding female infertility disorder, it is necessary to seek out alternative novel sources of stem cells (more supportive to female reproductive system) rather than traditional applied common sources for stem cells therapy.

### EnMSCs

Since EnMSCs are a promising candidate for treating infertility disorders due to their ability of self-renewal, high proliferation, and differentiation (60). It has been found that basal layer of the endometrium is an ample source EnMSCs; however, menstrual blood has also been indicated to contain EnMSCs, known as menstrual derived endometrial stem cells (MenSCs). Hence, representing a novel and promising source of MSCs to treat infertility in women and drawed the attention of clinicians as well as researchers (9).

Due to their convenient harvesting procedure with a high proliferation rate using noninvasive techniques, EnMSCs present no risk of rejection by the autoimmune system (61, 62). While EnMSCs have also been indicated to improve the proliferation and growth of injured endometrium by minimizing inflammation and fibrosis by elevating various growth differentiation factors 5 and signaling pathways (63, 64). EnMSCs, and hormonal stimulation, could preeminently restore the fertility potential in women affected by intrauterine synechiae (65), thus suggesting the significance of a combined treatment strategy.

Accordingly, a recent study has found that Yazd endometrial MSCs can not only increase, but also possess the potential to differentiate into various types of cells which make these cells an attractive source for clinical application particularly for the cure of patoents suffering from uterine-factor based infertility (66). Moreover, further studies have indicated that angiogenic and anti-inflammatory factors are the major contributor to the therapeutic effects of endometrial or MenSCs in eliminating the detrimental effects of intrauterine synechiae in an experimental rodent model (67). It could be due to the ability of MenSCs to regulate the protein kinase B signaling pathways that not only aid in the survival of MenSCs (68) but also provide a suitable environment for implanting embryos (69). Moreover, it has also been demonstrated that MenSCs enhance the endometrial thickness by elevating the expression of cell cycle inducers and reducing the expression of DNA damaging factors, which ultimately aids in perceiving the pregnancy for patients suffering from a uterus disorder such as intrauterine synechiae (67, 70-71). Notably, another piece of evidence has suggested that MenSCs can cure premature ovarian failure (POF) in young female patients (with a history of receiving chemotherapy). Hence, this suggesting the significant reparation potential of endometrial or MenSCs. Nonetheless, the underlying molecular mechanism which aid EnMSCs in curing infertility in women remain elusive to date, therefore, a precise set of comprehensive study design is prerequisite in future (72).

## 5. Role of cell-free therapy and female infertility disorders

SCs possess a powerful paracrine effect (contributing to their therapeutic effects), which not only pave the way for developing novel techniques also enhance the regenerative potential of SCs (73, 74). Importantly, due to the presence of micro vesicles, exosomes, and a variety of signaling factors, SCs-derived secretomes is considered as an effective therapeutic options (75), with the ability to repair of damaged tissue even in the absence of parent cells (76). Intriguingly, recent studies have also reported the effectiveness of condition medium from different cell sources for improving female infertility treatments. For instance, for assisted reproductive technology, in vitro maturation has reported being improved by using conditioned media from different sources (in vitro maturation). A higher level of female germ cell markers and granulosa cells was observed in female stem cells exposed to cumulus cells condition medium (CCCM) (44). Moreover, granulosa CCM has also been shown to improve follicle development in primordial mice. As a result, this provides evidence that human cumulus cells condition medium can support the IVM of mouse GV oocytes when derived from cultures of adherent cumulus cells (77). The effects testicular CCM has also been evaluated in vitro from embryonic stem cells on the development of female germ cells (78-80). For instance, testicular CCM was found to increase oocyte maturation in both mice and buffalo by secreting a variety of growth factors which enhance in vitro oogenesis (80). Intriguingly, humans also exhibit these factors (81).

Further research into the paracrine properties of stem cells has revealed that exosomes derived from stem cells have therapeutic potential to cure infertility in women. Exosomes are considered an optimal source for research due to their convenient isolation method and no risk of tumor formation (82, 83). For instance, it would be interesting to add that not only human UC-MSCs themselves can aid in the recovery of perimenopausal rats by accelerating the release of cytokines (51), but a study examining rat model of preeclampsia has reported that the UC-MSC-derived exosomes remarkably ameliorate the placenta by improving its morphology and angiogenesis in a dose-dependent manner (84). Adipocyte-derived exosomes injections have been shown to enhance ovarian function by reducing apoptosis and increasing follicle counts (70). On the other hand, a study on mice model of POF (that have been exposed to chemotherapy) demonstrated that exosomes derived from human amniotic epithelial cells contain transferring miRNAs that potentially aid in the repair of damaged ovary (85, 86).

Moreover, the use of exosomes (secreted by MSC) is also considered an alternative strategy to overcome the limitations of stem cell exposure, thus determining the advantage of cell-free therapy (43) and cell therapy (87). Hence, despite these promising experimental data, exosomes derived from stem cells have no reported clinical trial for the treatment of women's infertility. Therefore, to determine the precise clinical outcome of exosomes-based therapy for the cure of infertility in women, further preclinical data is required to establish the base for clinical trials.

## 6. Constraints in the clinical application of MSCs therapy for infertility disorders

Although there are few registered clinical trials studies in the NIH clinical trial database (www.clinicaltrials.gov) that exhibit the complete therapeutic outcome of stem cell therapy, although very few clinical studies have used stem cells to treat female infertility. However, to date available clinical trial results have shown the effectiveness of stem cell-based therapy to reviving the function of the damaged ovary to improve the regulation of hormonal and menstruation function (88). Accordingly, we have attempted to summarize some important clinical trial studies evaluating the potential of stem cells to cure infertility in females (Table II). This suggests that all studies remained under investigation and could not reach phase III for their practical application.

Several factors could determine the slow progress of stem cell therapy towards practical application. However, appropriate selection of participants, inclusion, and exclusion criteria is an important factors for the positive outcome of clinical trials. Advancing the clinical translation of preclinical studies is mainly constrained by the exclusion of pregnant or lactating patients, patients suffering from ovaries or breast cancer, with autoimmune diseases, and those with ovarian diseases such as endometriosis (89). However, it could be due to ethical and clinical concerns in each country regarding the healthcare policy (90).

Besides, the efficient clinical translation of preclinical trials remained the major challenge. This suggests that due to the difference in the microenvironment of human and animal models, the majority of animal trial studies could not be efficiently translated into human patients. Inadequacy for clinical translation of preclinical trials (which mainly carried on animals models) can be attributed to the difference in intrinsic microenvironments between animals and humans (91). While another study has presented the risk of developing autoimmune responses after stem cell transplantation, this aspect needs further research (92). Hence, at the moment, it is pretty hard to assume whether stem cell therapy could be effective in curing infertility in women or not. Therefore, a more comprehensive research design and study plans are required, which could preferably benefit patients with minimum risk of an immune response. Besides, it is also important to overcome various risk factors associated with the clinical application of MSCs. This could be attributed by establishing the safety parameters for the clinical application of MSCs (43) and by designing a real-time professional set up in preclinical trials to determine its outcome on clinical system, yet it remained challenging to date (93).

**Table 2:** Summary of clinical trials using stem cell therapy to treat female infertility disorders


**Author, yr **	**ClinicalTrials.gov Identifier**	**Study type**	**Type of cell**	**Type of infertility disorder**	**Patient's demography**	**Delivery route**	**Dose**	**Study outcome**
** Valeria Muller,** **2017**	NCT03166189	Interventional phase-II	BM-MSC	Female with Ashermen syndrome and infertility of uterine origin	Female at the age of 20-44 yr	endometrial injection of cells	1 ml of suspension containing 5 million BM-MSCs	Open randomized trial of clinical efficiency and safety of cell product BM-MSC for reparative treatment of destructively changed endometrium in patients with repeated IVF failures
**Hesham** **Elshaer, 2014 **	NCT02043743	Interventional phases 1 and 2	huCART-meso cells	Female with POF	Female at the age of 18-40 yr	Intraovarian injection	3-5 million MSCs injected into ovarian tissue	Autologous stem cells transplantation in patients with idiopathic and drug-induced premature ovarian failure
**Guangzhi Liu,** **2022**	NCT03816852	Interventional phase 2	Human UC-MSCs	Female up to 19-40 yr (Adult)	POI or POF	Intravenous infusion	From a high dose of 9*10 ${}^{\wedge }$ 7 cells, 30 ml, to a low dose of 3*10 ${}^{\wedge }$ 7	The safety and efficiency study of MSCs in POI
**Stem Cells** **Arabia, 2018**	NCT03069209	Interventional phase 1 and phase 2	Human BM-MSC	Females between the age of 20 to 39 yr	POF	Intraovarian transplantation	6*10 ${}^{\wedge }$ 7 cells, 30 ml	Autologous BM-MSC transplantation in patients with POF) aids folliculogenesis, normalizes the FSH level, and pregnancy occurred within 12 months of follow up
**Hongmei Wang,** **2021**	NCT03877471	Interventional	Embryonic stem cell-derived MSC-like cell transplantation directly into bilateral ovaries	Females up to the age of 40	POI	Ovary injection with MSC-like cells through transvaginal ultrasound	Patients received from low dose of 0.2 × 107 to a high dose of (1.0 × 107) cells	MSCs - like cell transplantation in women with primary ovarian insufficiency
BM-MSC: Bone marrow-derived mesenchymal stem cells, IVF: In vitro fertilization, huCART-meso cells: Human chimeric antigen receptor-modified T-meso cells, POF: Premature ovarian failure, MSCs: Mesenchymal stem cells, UC-MSCs: Umbilical cord mesenchymal stem cells, POI: Premature ovarian insufficiency, FSH: Follicle stimulating hormone								

## 7. Challenges and future perspective for application of stem cell therapy infertility disorder

The fact that engineered MSCs combined with the scaffold technique are considered a promising candidate to cure infertility-related disorders in females (43). However, therapeutic application of stem cell therapy remained restricted to few countries due to several technical and ethical limitations (89). Thus, this section will determine the challenges and some potential novel strategies to overcome these challenges. Besides, optimizing extraction and transplantation method of stem cells also remained the major concern for its therapeutic application (92). Besides, the route for administration of stem cell injection is also an important factors effecting the outcome of stem cell therapy (93). For instance, direct administration of stem cells into the ovaries is applied when the target is to restore ovarian function, while intravenous (i.v.) or intraperitoneal (i.p.) that allows fair distribution of stem cells through blood strea, can be used to evaluate the effects of stem cell transplantation for multiple organ system (89).

To overcome the limitations of stem cell exposure, recently, cell-free therapy has been developed (43) and reported to hold several advantages in cell therapy (88). For this purpose, the most practical approach is using exosomes (secreted by MSC), as discussed in the previous section. In addition to cellular therapy, the therapeutic sequel of stem cell treatment can also be improved by combined therapeutic strategies. For instance, biomaterials are increasingly being integrated for fertility disorders, not only to reduce the shear stress caused by stem cell injection but also enhance the probability of cell survival after administration (93). It has been demonstrated that the combined use of collagen scaffolds and stem cells aids in the rapid spread of stem cells to the targeted tissues or organs and enhances the probability of transplanted cells survival at the initial phase of transplantation in-vivo (43). Consistently, its has been shown in an experimental study that combined application of collagen scaffold with UC-MSCs in patients suffering from POF exhibited the successful activation of follicles in dormant ovaries (43). Although integrated therapeutic strategies are considered a potential candidate for developing a safe and efficient therapeutic strategy to overcome the clinical challenges associated with infertility disorders, precise understanding of the signaling mechanism remained elusive. Nonetheless, stem cells-derived exosomes and their vital content, such as miRNAs have also been suggested as the promising therapeutic candidate in the treatment of various ovarian dysfunction, particularly via folliculogenesis and genetic stability and vascular formation (91). However, further studies are required to unveil the exact mechanism. Taken together, despite of these novel molecular therapeutic modalities, further advent in the research and comprehensive knowledge of the underlying molecular mechanism is prerequisite to treat female fertility disorders.

## 8. Conclusions 

Developing an efficient therapeutic strategy based on the regenerative properties of stem cells holds the confidence and hope of scientists, clinicians, and patients suffering from fertility disorders. However, to obtain significant breakthroughs for the cure of infertility in women and for successful clinical translation of precise and well-designed study plan (from the isolation of stem cells or stem cell-derived molecules to informed, voluntary consent and model of cell delivery including) is essentially required for the success of initial clinical trials in the light of previously reported studies to overcome the limitations. Taken together, the present study attempted to present an overview of previous continued attempts and research studies to sort out the complex web of stem cell-derived therapeutic strategies and their role in treating female fertility disorders.

##  Conflicts of Interest

The authors declare that they have no competing interest.
